# Effect of Kimchi Fermentation on Oxalate Levels in Silver Beet (*Beta vulgaris* var. cicla)

**DOI:** 10.3390/foods3020269

**Published:** 2014-04-23

**Authors:** Yukiko Wadamori, Leo Vanhanen, Geoffrey P. Savage

**Affiliations:** Food Group, Department of Wine, Food and Molecular Biosciences, Lincoln University, Lincoln 7647, Canterbury, New Zealand; E-Mails: leo.vanhanen@lincoln.ac.nz (L.V.); Savage@lincoln.ac.nz (G.P.S.)

**Keywords:** total, soluble and insoluble oxalates, kimchi, lactobacilli, calcium

## Abstract

Total, soluble and insoluble oxalates were extracted and analyzed by high performance liquid chromatography (HPLC) following the preparation of kimchi using silver beet (*Beta vulgaris* var. cicla) stems and leaves. As silver beet contains high oxalate concentrations and consumption of high levels can cause the development of kidney stones in some people, the reduction of oxalate during preparation and fermentation of kimchi was investigated. The silver beet stems and leaves were soaked in a 10% brine solution for 11 h and then washed in cold tap water. The total, soluble and insoluble oxalate contents of the silver beet leaves were reduced by soaking in brine, from 4275.81 ± 165.48 mg/100 g to 3709.49 ± 216.51 mg/100 g fresh weight (FW). Fermenting the kimchi for 5 days at 19.3 ± 0.8 °C in 5 L ceramic jars with a water airtight seal resulted in a mean 38.50% reduction in total oxalate content and a mean 22.86% reduction in soluble oxalates. The total calcium content was essentially the same before and after the fermentation of the kimchi (mean 296.1 mg/100 g FW). The study showed that fermentation of kimchi significantly (*p* < 0.05) reduced the total oxalate concentration in the initial mix from 609.32 ± 15.69 to 374.71 ± 7.94 mg/100 g FW in the final mix which led to a 72.3% reduction in the amount of calcium bound to insoluble oxalate.

## 1. Introduction

Kimchi, a fermented traditional food has been made in Korea for several hundred years [1]. It is a method of preserving food for use in the winter and it is gaining popularity as a functional food because of the phytochemicals formed during the fermentation process [2]. It is typically consumed raw and served as a side dish with practically every meal. The most popular kimchi in Korea, Baechu kimchi, is made principally from Chinese cabbage [3] although almost all vegetables cultivated in Korea have been used in kimchi preparation [2]. Other ingredients are commonly added and these vary between producers, for example, red pepper powder, ginger, garlic and radish [1]. Other minor ingredients that can be included are Indian mustard greens, carrots and dropwort. Seasoning ingredients, green onions, Chinese leeks, onions, pine nuts, gingko nuts and sugar, are also added in variable amounts. It is also common to add fish sauce made from shrimps, oysters, Alaskan pollack, squid, flounder or yellow corvine [3]. The beneficial effects of kimchi on human health may be derived from the nutrients in kimchi, such as vitamins, minerals and phytochemicals present, in the ingredients used to make the kimchi or the fermentation products produced by the lactic acid bacteria [4].

The main ingredient of traditional kimchi, Chinese cabbage, is cut and soaked in a 10% to 15% salt solution for several hours until the leaves and stems become soft. The salted Chinese cabbage is then washed under running tap water and thoroughly drained. The seasonings are prepared and chopped and then stuffed between the drained cabbage leaves. The stuffed Chinese cabbage is then packed into a traditional container, a ceramic jar with a lid, and stored in a cool place. Traditionally, this jar was buried in the ground for days to months depending on the environmental temperature. Kimchi is processed in a traditional ceramic jar called an onggi and it is stored in special refrigerators which can control the optimum temperature of the fermentation [4]. Jeong *et al.* [5] showed that kimchi fermented in an onggi had higher nutritional profiles than when it was fermented in polyethylene, polypropylene, stainless steel or glass containers [5].

The microorganisms which exist in the raw materials and are found in fermented kimchi, are mostly lactic acid producing bacteria and yeasts [3]. The most important feature of kimchi for human health is that it contains very high levels of lactic acid-producing bacteria (10^8^ to 10^9^ CFU/g) [6] such as *Leuconostoc mesenteroides*, *Lactobacillus brevis*, *Lactobacillus plantarum*, *Pediococcus cerevisiae*, *Streptococcus faecalis*, *Enterococcus faecalis*, *Pediococcus pentosaceous*, *Weissella koreanis* and *Lactobacilli* spp. [7,8,9]. *Leuconostoc mesenteroides* and *Lactobacillus plantarum* are found in the largest numbers. Total aerobic bacteria and fungi are reduced during kimchi fermentation and yeasts increase under the low temperature conditions [7]. Temperature is the most crucial factor in determining the balance of microbial populations during fermentation [10]. Studies have identified a range of important factors, such as the final acidity should fall between 0.6% and 0.7% and the pH should be between 4.2 and 4.3 for optimum consumer acceptance [7,11,12,13].

Oxalate is found in many kinds of edible plants with variable concentrations [14,15,16]. As consumption of additional oxalate in the diet can cause the development of kidney stones in susceptible people it is important to identify high oxalate containing foods and, if possible, reduce these levels by processing. As kimchi can be made from a range of vegetables and fruits, some kimchi may be produced from plants and spices which contain higher levels of oxalate. In addition, oxalates occur in two forms in plants, water soluble oxalate bound to Na^+^ or K^+^, or water insoluble oxalate bound to divalent ions such as Ca^2+^ and Mg^2+^. Simpson *et al.* [17] presented a pH speciation diagram for oxalic acid which showed in an ideal solution that the two forms of oxalate are affected by the pH of the medium. At a pH lower than six, the proportion of fully deprotonated divalent oxalate ions (C_2_O_4_^2−^) decreases markedly with a correspondingly reduced potential for binding with divalent mineral cations (especially Ca^2+^) to form insoluble oxalates. Therefore, a reduction in the pH of kimchi during the fermentation will have an effect on the proportions of soluble and insoluble oxalates in the final kimchi mix. The overall effect will be to increase the soluble oxalate content of the fermenting mix. This would provide increased levels of soluble oxalates that could then be used by the anaerobic bacteria that form a large proportion of the fermentation biomass in the kimchi. Some lactic acid producing bacteria have been reported to break down oxalate and use it as a carbon and energy source *in vitro* [18,19]. *Lactobacillii plantarum* is the dominant strain of bacteria commonly found in kimchi fermentations and it has been shown that two strains of *Lactobacillus acidophilus* and one strain of *Lactobacillii plantarum* also have the ability to degrade oxalate [20]. 

The leaching of soluble oxalate during washing and soaking in brine solution may reduce the oxalate content of kimchi. In addition, small amounts of liquid removed after fermentation may contain leached oxalates and this fraction is not consumed. 

The stems and leaves of silver beet are consumed widely in New Zealand and could be an inexpensive and interesting alternative to Chinese cabbage for communities in New Zealand who regularly consume kimchi. However, silver beet is a high-oxalate containing vegetable and needs to be eaten in small quantities by people predisposed to oxalate kidney stones. It is known that addition of calcium sources can reduce the amount of soluble oxalate available for absorption from the gastrointestinal tract but the effect of fermentation has not been previously studied [21,22]. 

The aim of this study was to investigate the use of the leaves and stems of silver beet, a non-traditional vegetable, to make kimchi. This study involved the measurement of the effect of soaking silver beet stems and leaves in brine and the effect of low pH and microbial fermentation on the oxalate content in silver beet during the production of traditional kimchi. 

## 2. Experimental Section

### 2.1. Preparation of Kimchi

Ten kilograms of fresh, fully grown silver beet plants (*Beta vulgaris* var. cicla), seven onions (*Allium cepa*), three apples (*Malus pumila*), one head of garlic (*Allium sativum*) and one piece of fresh ginger (*Zingiber officinale*) were purchased from a local grower (Crazy Dave, Christchurch, Canterbury, New Zealand). Ten large white radishes (*Raphanus sativus*) and seven bunches of Chinese chives (*Allium tuberosum*), all grown in New Zealand, were purchased from a local Chinese shop (Chinese market, Christchurch, Canterbury, New Zealand). Salt (Cerebos Skellerup Ltd., East Tamaki, Auckland, New Zealand) and red chilli powder (*Capsicum annuum*, Ducsan, Jung-gu, Seoul, South Korea), fermented small shrimps (packed in Shirley Fish Market, Christchurch, New Zealand, produced in Korea), Korean fish sauce (Chung Jung One, Samseong-dong, Gangnam-gu, Seoul, Korea), sugar (Chelsea Sugar Company, Birkenhead, Auckland, New Zealand) and glutinous rice flour (Samutsakorn, Mahachai, Samut Sakhon, Thailand) were all purchased from a local Korean shop (Kosco, Christchurch, Canterbury, New Zealand).

Ten kilograms of silver beet leaves and stems were washed under running tap water and chopped into 40 mm pieces. The prepared silver beet was then soaked in 20 L of 10% salt water and allowed to soak for 2 h at room temperature (19.3 ± 0.8 °C) then 9 h in the fridge at 5.4 °C. The salted silver beet leaves and stems were squeezed by hand to remove excess water and then washed gently under running tap water and squeezed again to remove excess water.

White radishes (3.22 kg) were cut into fine thin strips. Chinese chives (0.58 kg) were cut into 30 mm pieces. Seven onions (1228 g), three small apples (192 g), fresh ginger (65 g) and one garlic clove (15 g) were chopped into small pieces and then ground together using a mixer (Multipro, Kenwood, Japan). Glutinous rice powder (100 g) and water (1.33 kg) were mixed and heated in a saucepan until gelatinized. The chopped and processed vegetables were pre-mixed with the cooled gelatinized rice, 980 g of chilli powder, 33 g of salt, 300 g of fermented small shrimps, 730 g of Korean fish sauce and 200 g of sugar. This mixture was then placed in a 40 L plastic flexi-tub food container. The salted silver beet leaves and stems were then added and thoroughly mixed by hand. The mixed ingredients were then placed into three traditional (five liter) Korean kimchi jars and a plastic film was placed over the top of the mixture to keep it moist. A weight and a cap were put on each jar and water was poured into the upper rim in order to prevent air entering the jars during fermentation. The jars were allowed to stand at room temperature (mean 19.3 ± 0.8 °C) for 5 days. 

### 2.2. Dry Matter and pH Determination

The dry matter (DM) content of each sample was determined by drying in an oven (Watvic, Watson Victor Ltd., New Zealand) to a constant weight at 105 °C [23]. The pH in the initial and the mix at the end of the 5 day fermentation period were measured at ten different points in the mix using a SevenEasy pH Meter (Mettler Toledo, GmbH, Schwerzenback, Switzerland). 

### 2.3. Total and Soluble Oxalate Analysis

Six representative samples of kimchi were collected from each jar before and after fermentation for oxalate analysis. The liquid remaining at the bottom of each jar was also sampled. For the extraction of the total oxalic acid, 0.2 g of ground oven-dried sample was weighed accurately into a 100 mL flask and 40 mL of 0.2 M HCl was added and the mixture was then incubated with shaking for 20 min at 80 °C. After cooling, each mixture was transferred to a 100 mL volumetric flask and made up to volume with 0.2 M HCl. Three extractions were carried out on each sample. Fifty milliliters of the aliquot was transferred in a 50 mL centrifuge tube then centrifuged at 3500 rpm for 15 min. The liquid sample was filtered through a 0.45 μm cellulose acetate syringe filter (Sartorius A.G., Göttingen, Germany) into a HPLC vial for analysis of the oxalic acid concentration. 

For the extraction of the soluble oxalic acid, the same procedure as followed as in the extraction for total oxalic acid except that nanopure water (18.2 Megaohm-cm, Arium 611 uv, Sartorius A.G., Germany) was used to extract the soluble oxalates.

The oxalic acid concentration of the extracts was determined using an HPLC method where a 20 μL of sample extract was analysed using a 300 × 7.80 mm Rezex ion exclusion column (Phenomenex Inc., CA, USA) using isocratic elution at 0.6 mL/min. with 0.025 M sulphuric acid (HPLC Grade, Baker Chemicals, Phillipsburg, NJ, USA) as the mobile phase [15]. The HPLC system consisted of a Spectra-Physics Isocratic pump and a Spectra-Physics UV/V detector set at 210 nm. Data capture was performed using PeakSimple version 3.59 (SRI Instruments, Torrance, CA, USA). The oxalate peak was identified by comparison of the retention time with an oxalate standard (99.999%, Sigma, St. Louis, MO, USA). Insoluble oxalate was calculated as the difference between the total oxalate (acid extract) and soluble oxalate (water extract) [24]. The oxalate results are presented as mg/100 g fresh weight (FW).

Standard curves, containing 2–20 mg oxalic acid/100 mL in water or 0.2 M HCl were prepared and used to quantify the soluble and total oxalic acid contents of the samples.

### 2.4. Total Calcium Analysis

Duplicate samples of oven dried kimchi were initially ground in a coffee mill (Sunbeam, model EM0400, China), then 0.3 g accurately weighed into a 75 mL Teflon PFA Kevlar-shielded digestion vessel (CEM Corporation, Matthews, NC, USA). To this, 5 mL of a 4:1 nitric acid (69%): hydrogen peroxide (Aristar grade, Merck Ltd., KGaA, Darmstadt, Germany) acid mixture was added. The digestion vessels were then capped and the oven dried kimchi samples were digested using a MARSXpress™ microwave digester (CEM Corporation, Matthews, NC, USA), programmed to ramp from ambient to 90 °C over 10 min then from 90 °C to 170 °C over 10 min where it was held for 10 min. Once cooled, 10 mL of deionized water (18.2 Megaohm-cm) was added to make a final volume of 15 mL. 

Mineral analysis was carried out on a Varian Axial 720 Inductively Coupled Plasma Optical Emission Spectrophotometer (ICP-OES, Varian, Palo Alto, CA, USA) with SP3 auto-sampler. Minerals were identified and quantitated using an ICP multi-element standard solution (CertiPUR, Merck, KGaA, Darmstadt, Germany) containing 23 elements or a single element standard, as required. Data and standard curves were processed using ICP-Expert™ II (Varian, Palo Alto, CA, USA). A water test standard and the ICP multi-element standard solution containing 0.50 μg/g of each element were run in triplicate with each batch to determine the standard error. The overall standard error was ±0.013 mg/kg. Limits of quantitation (LOQ) were performed using 10 times the standard deviation of the blank (5% nitric acid) for each mineral. Individual mineral LOQ values ranged from 0.12 to 12.24 μg/L, with a mean of 1.81 μg/L. The measurement of the total calcium in the kimchi mix and the calculation of the calcium content of the insoluble oxalate allowed the calculation of the proportion of insoluble calcium to total calcium content of the kimchi mix.

### 2.5. Statistical Analysis

All calculations were performed using Excel 2010. GenStat Release 12.2 for Windows 7 (VSN International Ltd., Hemel Hempstead, Hertfordshire, UK) was used to determine the accumulated analysis of variance. The mean values were compared using Fishers LSD method (*p* < 0.05).

## 3. Results and Discussion

The mean pH of the initial kimchi mix was 5.06 ± 0.307 and this reached 4.34 ± 0.042 after 5 days fermentation at 19.3 ± 0.8 °C. This decrease in pH was due to the formation of lactic acid by the bacteria. The dry matter content of the raw and processed silver beet leaves and stems, and the kimchi mixes are shown in Table 1. This shows that soaking silver beet leaves in salt water significantly reduced the moisture content of the leaves. The average dry matter of raw and processed silver beet leaves and stems, and the kimchi mixes, ranged from 7.1% in the raw leaves and stems of silver beet to 14.8% in kimchi mix at end of experiment.

**Table 1:** Dry matter content of the raw and processed silver beet leaves and stems, and the kimchi mixes (%).

Food sample	Mean dry matter content (%) (±SE)
Raw leaves and stems of silver beet	7.07 ± 0.09
After soaking in salt water	14.00 ± 0.65
Kimchi mix at start of experiment	13.44 ± 0.12
Kimchi mix at end of experiment	13.99 ± 0.14

The dry matter content of the kimchi increased during fermentation as some liquid was released from the kimchi mass. The total, soluble and insoluble oxalate contents of the raw and processed silver beet leaves and stems are shown in Table 2.

**Table 2:** Mean total soluble and insoluble oxalate contents of the raw and processed silver beet used to make kimchi (mg/100 g fresh weight).

Silver beet	Total oxalate	Soluble oxalate (% of total oxalate)	Insoluble oxalate
Raw leaves and stems of silver beet	4275.81 ± 165.48	1880.67 ± 378.95 (44.0%)	2395.15 ± 397.71
Raw leaves and stems of silver beet after soaking in 10% salt water	3709.49 ± 216.51	1811.68 ± 209.42 (48.8%)	1897.82 ± 207.29

The oxalate values of the raw commercially produced silver beet leaves and stems used in this experiment are higher than previously reported data [15,17] but it has been previously noted that the levels of oxalates in silver beet leaves are very variable [17]. Soaking the silver beet leaves and stems in brine for 11 h reduced total oxalate content on a wet matter basis by 13.2% with only a small reduction in the soluble oxalate content (3.67%). 

The oxalate content of the onions, apples, garlic, fish sauce, fermented shrimp, radishes and Chinese chives were measured but no oxalate was detected. The oxalate contents of fresh ginger and dry red chilli powder are shown in Table 3.

**Table 3:** Mean total soluble and insoluble oxalate contents of materials used to make kimchi. FW, fresh weight; DM, dry matter.

Food sample	Total oxalate	Soluble oxalate	Insoluble oxalate
Fresh ginger (mg/100 g FW)	1312.66 ± 30.99	1174.06 ± 65.43	138.60 ± 96.41
Red chilli powder (mg/100 g DM)	274.01 ± 9.8	277.22 ± 4.58	-

Total oxalate contents of the fresh ginger was very high and were similar to values reported in an earlier study [25] where dried ground ginger powder contained 1528 ± 92 mg/100 g of total oxalate and 1339 ± 38 mg/100 g of soluble oxalates.

Silver beet leaves and stems consisted of 56.7% of the total fresh kimchi mix and, therefore, supplied the greatest proportion of oxalates to the initial kimchi mix. Chilli powder and fresh ginger also supplied some oxalates although they only represented 5.6% and 0.4% respectively, of the total mix. The initial and final total, soluble and insoluble oxalate contents of the kimchi mixes are shown in Table 4. The total, soluble and insoluble oxalate contents of the kimchi were significantly reduced by 38.5%, 22.9% and 70.4%, respectively, following five days of lactic acid bacteria fermentation in kimchi jars at a mean temperature of 19.3 ± 0.8 °C. The proportion of soluble oxalate to total oxalate in the kimchi mix increased from 67.1% to 84.1%.

**Table 4:** Mean total soluble and insoluble oxalate contents of kimchi before and after fermentation (mg/100 g FW).

	Jar	Total	Soluble	Insoluble
			(% of total oxalate)	
Before fermentation	A	661.63 ± 6.6	446.38 ± 0.6	215.25 ± 6.0
	B	615.90 ± 11.7	407.20 ± 21.0	208.70 ± 27.3
	C	550.43 ± 4.2	372.20 ± 13.2	178.30 ± 15.5
Mean		609.32 ± 15.69^a^	408.58 ± 12.16^a^ (67.1%)	200.75 ± 10.23^a^
After fermentation	A	372.70 ± 17.9	299.30 ± 16.2	73.40 ± 26.5
	B	385.56 ± 9.5	323.97 ± 8.4	61.60 ± 14.6
	C	365.90 ± 13.7	322.35 ± 6.0	43.60 ± 11.3
Mean		374.71 ± 7.94^b^	315.19 ± 23.21^b^ (84.1%)	59.52 ± 10.54^b^

Mean values before and after fermentation with a different letter, differ significantly (Fishers LSD, 95% level of confidence).

The mean liquid leachate remaining in each of the jars after five days of fermentation was 230 ± 17.8 mL which was 1.58% of the total kimchi mix in the three jars. As this leachate is not consumed, the removal of this liquid results in a small loss of oxalates from the kimchi available for consumption. The mean total and soluble oxalate contents of this fraction were, respectively, 353.78 ± 11.79 and 322.88 ± 8.60 mg. 

It is most interesting to note that large reductions in total, soluble and insoluble oxalates occurred during the fermentation of kimchi (38.5% for total oxalate, 22.9% for soluble oxalate and 70.4% for insoluble oxalate). This is the first time this has been observed during the fermentation of kimchi and is presumably the result of lactic acid bacteria fermentation that occurred when all the oxygen was removed from the atmosphere of the jar during the initial stages of the microbial fermentation of kimchi. There are two reasons for the reduction of oxalate contents during the lactic acid fermentation in the kimchi: (1) as the pH was reduced, the form of oxalate would change from insoluble oxalate bound to calcium ions to soluble oxalate [17]; and (2) this, then, will increase the soluble oxalate content of the liquid fraction of the kimchi mix and the increased soluble oxalate content could then be used as an energy source by the oxalotrophic bacteria present in the kimchi fermentation reducing both the total and the soluble oxalate contents of the final product in the process. This supports the observations made in an earlier study where *Lactobacillii plantarum,* was reported to be found in the kimchi in large numbers [7,8,9] and were found to degrade oxalate in other studies [20].

The total calcium content of the kimchi mix before and after fermentation was essentially the same, mean 296.0 mg calcium/100 g FW (Table 5). If it is assumed that insoluble oxalate is predominantly calcium oxalate [17] then it is possible to calculate the amount of calcium unavailable in this molecule and compare this with the total calcium in the kimchi mix. Table 4 shows that there was a 70.4% reduction in the insoluble oxalate content when the values found in the initial and final kimchi mixes were compared. This corresponds to a reduction calcium bound in insoluble oxalate (Table 5). The pH of the kimchi mix reduced from an initial 5.06 to 4.34 when fermentation was completed and the speciation diagram of oxalate with pH [17] shows that this pH change will result in a large reduction in the potential of oxalate to bind to divalent cations (especially calcium). Overall, fermentation led to 72% reduction of calcium bound in insoluble oxalate.

**Table 5:** Mean total calcium, calculated calcium bound in insoluble oxalate and % bound calcium in kimchi before and after fermentation (mg/100 g FW).

Kimchi	Total calcium (mg/100 g FW)	Calcium in insoluble oxalate (mg/100 g FW)	Insoluble calcium/total calcium (%)
Before fermentation	286.94 ± 9.23	62.79 ± 3.57	21.84 ± 0.56
After fermentation	305.17 ± 7.73	18.61 ± 2.72	6.06 ± 0.74

## 4. Conclusions

The use of silver beet to prepare kimchi has not been considered before but this experiment has shown that considerable reductions in the oxalate content do occur during the fermentation process. This experiment confirms that lactic acid bacteria responsible for traditional kimchi fermentation have oxalotrophic activity. Although the soluble oxalate as a proportion of total oxalate increased, the absolute amount in the fermented kimchi available for absorption decreased. 
